# A rare case of primary intracranial myxoma in a 10-year-old male: Case report and literature review

**DOI:** 10.1016/j.radcr.2026.07.008

**Published:** 2026-08-01

**Authors:** Moustafa A. Mansour, Reem W. Malaeb, Afnan Morad, Rawad Hakim, Hamdi Nabawi Mostafa, Mohamed Said Elsanafiry

**Affiliations:** a Department of Neurosurgery, Nasser Institute for Research and Treatment, Cairo, Egypt; b Department of Health Professions, Faculty of Health Sciences, American University of Beirut, Beirut, Lebanon; c Faculty of Medicine and Medical Sciences, University of Balamand, Balamand, Lebanon; d Department of Neurosurgery, Faculty of Medicine, Misr University for Science and Technology, Giza, Egypt; e Department of Neurosurgery, Faculty of Medicine, Menoufia University, Menoufia, Egypt

**Keywords:** Primary intracranial myxoma, Pediatric neurosurgery, Dural-based tumor, Seizure, Myxoid neoplasm

## Abstract

Primary intracranial myxoma (PIM) is an exceptionally rare, benign mesenchymal neoplasm arising *de novo* within the central nervous system, distinct from the more common cardiac myxoma with central nervous system embolization. We report the case of a previously healthy 10-year-old male who presented with a first-time witnessed generalized tonic–clonic seizure lasting approximately 90 seconds, with clinical features suggesting a focal onset (head version to the left and asymmetric tonic posturing of the right arm). Postictally, the patient regained full consciousness after 15 minutes with no focal neurological deficits. Neuroimaging revealed an extra-axial, dura-based lesion in the left frontal lobe. Preoperative magnetic resonance imaging (MRI) demonstrated a well-defined, T1-hypointense, T2/FLAIR-hyperintense lesion with strong homogeneous contrast enhancement, insinuating between the superior and middle frontal gyri without significant mass effect. The patient underwent gross total resection via a pterional approach, revealing a well-capsulated, gelatinous, multilobulated tumor. Histopathological examination confirmed a connective tissue lineage neoplasm with stellate cells embedded in a myxoid matrix, consistent with a myxoma. Post-operative echocardiography and cardiac MRI ruled out a cardiac primary source, confirming the diagnosis of PIM. The patient was initiated on levetiracetam 20 mg/kg/day postoperatively, which was successfully tapered and discontinued after 3 months. The patient had an uneventful recovery with no seizure recurrence at 18-month follow-up. This case underscores PIM as a diagnostic consideration for dura-based, well-circumscribed intracranial lesions in children. A multimodal approach combining characteristic neuroimaging, meticulous histopathology, and thorough systemic investigation to exclude a cardiac source is essential for accurate diagnosis and management, which yields an excellent prognosis following complete surgical resection.

## Introduction

Myxomas are benign, slow-growing tumors of mesenchymal origin, most frequently encountered in the cardiac atria, with other common sites including skin and jawbone [1]. While cardiac myxomas are the most common primary heart tumor in adults, their central nervous system (CNS) involvement is typically secondary to embolic events or, less commonly, metastatic deposits [2,3]. In stark contrast, a primary intracranial myxoma (PIM) is defined as a myxoma arising *de novo* within the CNS parenchyma or meninges, with no evidence of a primary tumor elsewhere [4]. PIMs predominantly affect children and young adults, although cases have been reported across a broad age spectrum. Owing to their exceptional rarity, no reliable incidence data exist; fewer than several dozen well-documented cases have been reported in the literature, with no consistent sex predilection has been demonstrated [5]. Clinical manifestations are largely determined by tumor location and commonly include headache, seizures, focal neurological deficits, visual disturbances, or symptoms related to mass effect. Laboratory investigations are generally nonspecific and diagnosis is achieved through integration of suggestive neuroimaging features, definitive histopathological examination, and rigorous exclusion of an extracranial primary source, particularly a cardiac myxoma. Surgical management typically consists of gross total resection through a tailored craniotomy approach, which remains the cornerstone of treatment and is associated with favorable long-term outcomes.

We present a rare pediatric case of a histologically confirmed PIM presenting with a new-onset seizure, detailing its clinical, radiological, surgical, and histopathological features, followed by a review of pertinent literature. Beyond documenting a rare pediatric case, this report contributes to the growing body of evidence characterizing the radiological–pathological correlation of primary intracranial myxoma. The combination of marked T2/FLAIR hyperintensity, homogeneous enhancement, absence of diffusion restriction, and minimal perilesional edema reflected the tumor’s abundant myxoid matrix and low cellularity. Furthermore, we emphasize the importance of a systematic diagnostic pathway integrating advanced neuroimaging, histopathological assessment, and comprehensive cardiac evaluation to distinguish true primary intracranial myxoma from secondary manifestations of cardiac myxoma.

## Case presentation

A 10-year-old, previously healthy male presented to the emergency department following a witnessed generalized tonic–clonic seizure, which was his first such episode. The seizure was reported to last approximately 90 seconds. Clinical features suggesting a focal onset included head version to the left and asymmetric tonic posturing of the right arm prior to generalization. The seizure did not meet criteria for status epilepticus. There was no significant past medical or family history. Postictally, the patient regained full consciousness after approximately 15 minutes of confusion, with no Todd's phenomenon or focal neurological deficits. Neurological examination was entirely normal, with no focal deficits or systemic manifestations.

In the emergency department, the patient received intravenous lorazepam 0.1 mg/kg for seizure termination (given that the seizure had already stopped spontaneously upon arrival, this was administered prophylactically to prevent recurrence) and a loading dose of levetiracetam 20 mg/kg intravenously. An electroencephalogram (EEG) performed within 24 hours of admission demonstrated a normal background rhythm with no epileptiform discharges or focal slowing. Given the presence of a structural lesion on neuroimaging, the patient was admitted for further evaluation and surgical planning.

### Imaging findings

**Fig. 1:**
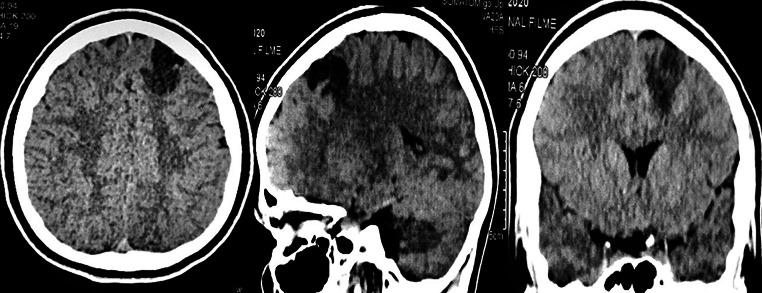
Axial, coronal, and sagittal noncontrast CT images demonstrate a well-defined, hypodense lesion in the left frontal lobe. The lesion is situated between the superior and middle frontal gyri, is in contact with the dura mater, and indents the cortical surface without mass effect.

An initial non-contrast computed tomography (CT) scan of the brain revealed a well-defined hypodense lesion in the left frontal lobe, situated between the superior and middle frontal gyri, attached to the dura mater without mass effect (Fig. 1). For further characterization, brain MRI was performed. The lesion was extra-axial, insinuating between the gyri while maintaining a clear plane with the underlying brain parenchyma.

**Fig. 2:**
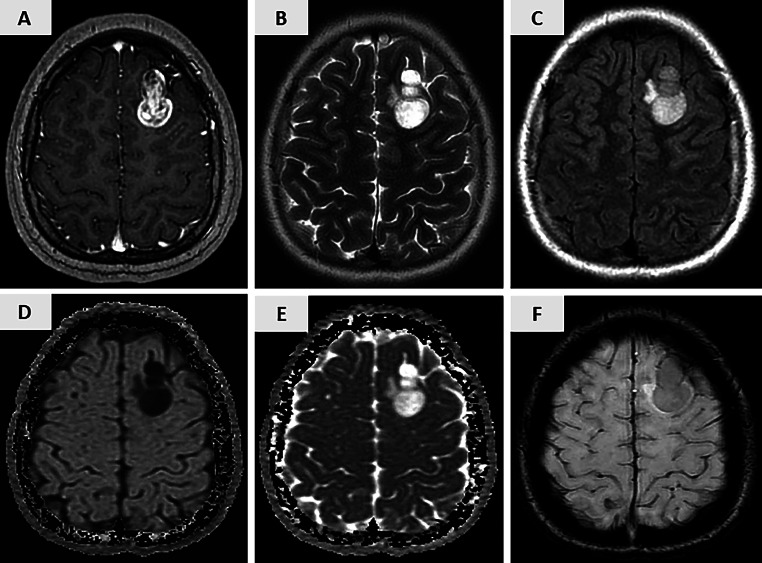
**MR imaging characteristics of the left frontal lesion.** The lesion abuts the dura mater at its site of apparent implantation and insinuates between the superior and middle frontal gyri, maintaining well-defined margins. (A) Postcontrast T1-weighted image shows strong, homogeneous enhancement. (B) T2-weighted and (C) FLAIR images demonstrate homogeneously high signal intensity with subtle internal signal variation, suggesting variable hydration. There is minimal adjacent white matter edema but no mass effect, consistent with slow growth. (D) Diffusion-weighted imaging (DWI) and (E) apparent diffusion coefficient (ADC) maps show no restriction of water diffusion, supporting the benign, low-cellularity nature of the lesion, consistent with the abundant myxoid matrix and lack of dense cellular packing characteristic of myxomas. (F) SWAN sequence reveals no signal loss, indicating absence of calcification or hemorrhage.
• T2-weighted and FLAIR images: Demonstrated homogeneously high signal intensity with subtle internal variation, suggestive of variable hydration (Fig. 2B and C). Minimal perilesional white matter edema was noted, consistent with slow-growing, non-infiltrative behavior.
• Diffusion-weighted imaging (DWI) and ADC maps: Showed no restriction of water diffusion (Fig. 2D and E), supporting the benign, low-cellularity nature of the lesion.
• SWAN sequence: Revealed no evidence of calcification or hemorrhage.

• T1-weighted (T1W) images: The lesion was hypointense.
• Contrast-enhanced T1W images: Showed strong, homogeneous enhancement (Fig. 2A).

These findings were suggestive of a benign, extra-axial, dura-based neoplasm such as a meningioma, schwannoma, or, given its gelatinous appearance on sequence analysis, a myxomatous lesion.

### Surgical intervention

The patient underwent a left pterional craniotomy. Intraoperatively, 3 well-capsulated, lobulated lesions with a gelatinous consistency were identified. They were firmly attached to the overlying dura by a fibrous band. Using Dowling’s technique with normal saline hydro-dissection and microsurgical dissection, the tumors were meticulously separated from the adjacent brain parenchyma and excised in *toto*.

### Histopathological examination

**Fig. 3:**
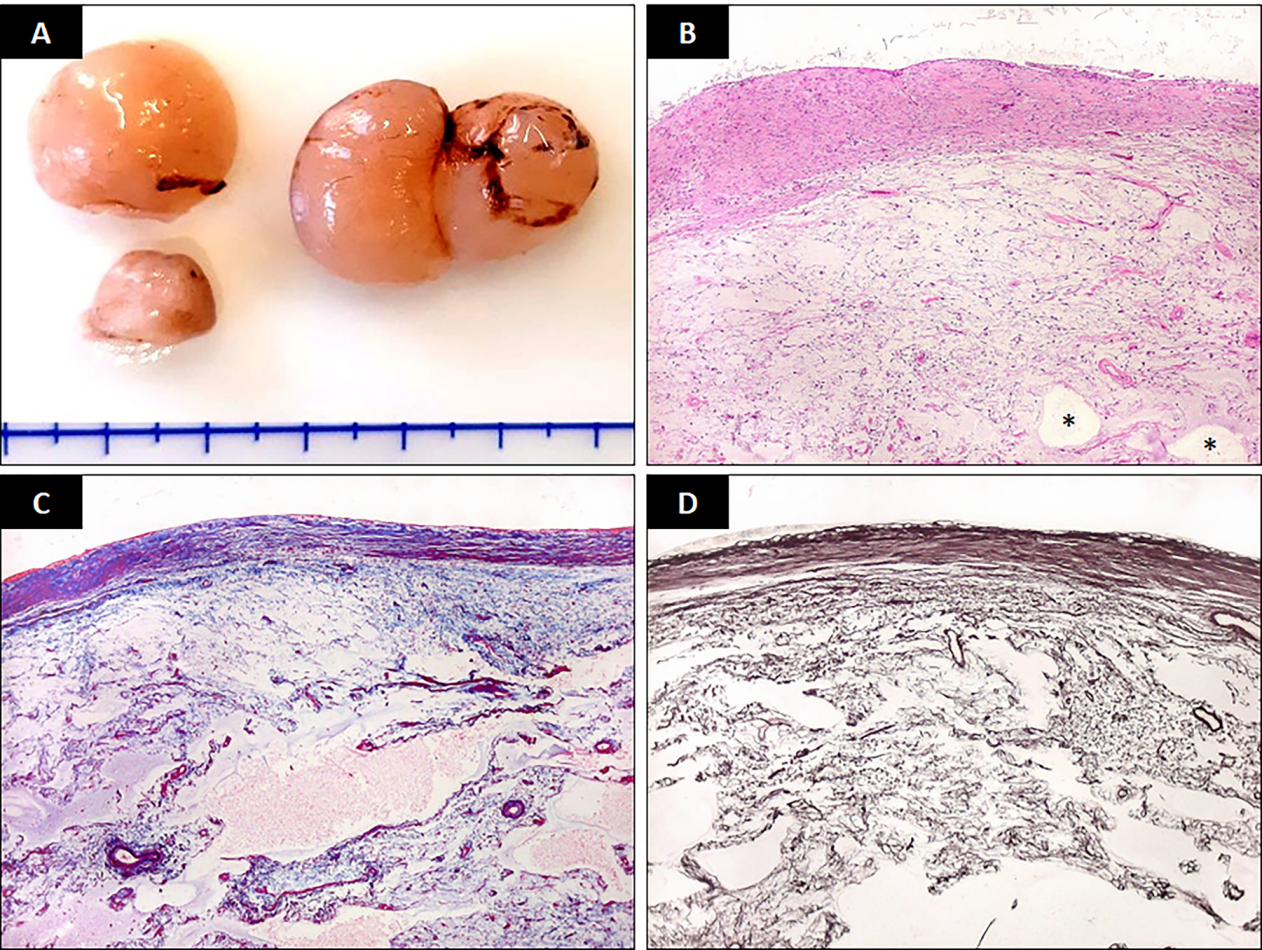
**Macroscopic and histopathological features of the resected lesion.** (A) Gross surgical specimen shows 3 small, well-defined, soft tissue nodules with a homogeneous, gelatinous cut surface. The largest fragment corresponds to the area of dural adhesion. (B) Hematoxylin and eosin (H&E) stain reveals a connective tissue neoplasm composed of large, pleomorphic cells with abundant basophilic cytoplasm and elongated tapering processes. Nuclei are irregular with poorly distributed chromatin but no significant atypia or hyperchromasia. The cells are dispersed within a highly hydrated, myxoid matrix containing delicate and thick collagen fibers arranged randomly. A fibrous capsule surrounds the tumor. In some areas, the myxoid matrix forms sparsely cellular lakes (asterisk). (C) Masson’s trichrome stain highlights collagen fibers in blue and cellular elements in red, demonstrating abundant collagen intimately associated with neoplastic cells. Also noted are lakes of amorphous myxoid material and thick-walled blood vessels. (D) Reticulin stain (Gomori’s silver impregnation) elegantly depicts the delicate network of reticulin fibers supporting the tumor cells, with probable partial impregnation of collagen fibers.

Grossly, the specimens were soft tissue nodules with a homogeneous, glistening, gelatinous cut surface (Fig. 3A). Hematoxylin and Eosin (H&E) staining revealed a connective tissue neoplasm composed of large, pleomorphic stellate cells with abundant basophilic cytoplasm and elongated tapering processes (Fig. 3B). Nuclei were irregular with poorly distributed chromatin but without significant atypia or mitotic activity. The neoplastic cells were dispersed within an abundant, highly hydrated myxoid matrix containing a random arrangement of delicate and thick collagen fibers. A fibrous capsule surrounded the tumor, and in areas, the matrix formed sparsely cellular “lakes.” Special stains provided further characterization: Masson’s trichrome highlighted the abundant blue-stained collagen fibers interspersed with red-stained cellular elements (Fig. 3C), while Gomori’s reticulin stain demonstrated a delicate network of reticulin fibers supporting the tumor cells (Fig. 3D). These features were diagnostic of a myxoma.

### Postoperative course and systemic work-up

The patient’s postoperative course was uneventful, and he was discharged fully conscious with no neurological deficits. Given the histological diagnosis of myxoma—most commonly a cardiac primary—a comprehensive cardiac evaluation was performed. Both transthoracic echocardiography and cardiac MRI showed no evidence of intracardiac mass or tumor, confirming the diagnosis of a Primary Intracranial Myxoma. Post-operatively, the patient was initiated on levetiracetam for seizure prophylaxis at a dose of 20 mg/kg/d (10 mg/kg twice daily) administered orally. The medication was continued for 3 months postoperatively. Subsequent tapering was performed with a 25% dose reduction every 2 weeks, guided by the patient's seizure-free status and a normal follow-up EEG showing no epileptiform activity. No adverse effects attributable to levetiracetam were observed during the treatment period. At 18-month follow-up, the patient remains seizure-free with no evidence of tumor recurrence on surveillance imaging.

## Discussion

Primary intracranial myxoma is an exceptionally rare, benign mesenchymal neoplasm that arises de novo within the cranial cavity, distinct from the far more common cardiac myxoma with secondary CNS involvement [[6], [7], [8]]. Since Virchow's first description of myxomatous tissue in 1871, these tumors have been most frequently associated with the heart, where they are well known to cause neurological complications via embolization or metastatic spread [4]. The extreme rarity of PIM and its nonspecific clinical and radiological presentation make preoperative diagnosis a significant challenge.

Our case of a 10-year-old male presenting with a new-onset generalized tonic–clonic seizure is consistent with the reported literature, where seizure is one of the most frequent initial manifestations of PIM. The detailed seizure semiology in our case—including head version and asymmetric posturing suggesting focal onset—highlights the importance of thorough clinical documentation in pediatric patients with structural brain lesions. The normal EEG findings, while reassuring, do not exclude the possibility of future seizure activity, underscoring the rationale for postoperative antiseizure prophylaxis in this population. The pediatric age of our patient aligns with the observed predilection for children and young adults, although cases have been reported across a wide age range (Table 1). Anatomically, PIMs demonstrate variable intracranial locations but frequently exhibit an extra-axial, dura-based attachment, supporting a proposed meningeal or perivascular mesenchymal origin [5,8]. In our patient, the lesion was situated in the left frontal lobe, insinuating between the superior and middle frontal gyri with clear dural contact—a feature that closely mirrors many previously documented cases [2,5,9,10,12,13].

**Table 1:** Summary of reported primary intracranial myxoma cases^a^.

Study	Age/Sex	Location	Presentation	Treatment	Outcome
Nagatani et al. [9]	63/F	Pituitary fossa	Visual disturbance	GTR	Recurrence at 2 y
Klein et al. [10]	NR	Posterior fossa	NR	GTR	NR
Graham et al. [5]	15/F	Frontal	Seizure	GTR	No recurrence (12 mo)
Osterdock et al. [11]	17/M	Temporal bone	Hearing loss	GTR	No recurrence (NR)
Menon et al. [2]	30/M	Parietal	Seizure	GTR	No recurrence (24 mo)
Kawatra et al. [1]	8/F	Occipital	Headache	GTR	No recurrence (12 mo)
Oruckaptan et al. [14]	42/F	Skull base	Headache	GTR	No recurrence (18 mo)
Powers et al. [12]	18/M	Parafalcine	Seizure	GTR	No recurrence (12 mo)
Yin et al. [13]	35/M	Skull base	Visual disturbance	GTR	No recurrence (36 mo)
Ryu et al. [15]	46/F	Ethmoid sinus	Epistaxis	GTR	No recurrence (24 mo)
Present case (2025)	10/M	Frontal	Seizure	GTR	No recurrence (18 mo)

GTR, gross total resection; NR, not reported.

a Literature search cutoff date: March 2025.

An intriguing observation from the available literature is the relatively frequent occurrence of PIM in pediatric and young adult patients. Although the biological basis remains uncertain, several authors have proposed that these tumors may arise from primitive mesenchymal cells or embryonic mesenchymal rests within the meninges and adjacent connective tissues that persist during development [5]. Such a developmental origin may partially explain the younger age distribution reported in many cases. However, given the extremely limited number of published cases, definitive conclusions regarding age-related susceptibility cannot yet be established.

Radiologically, PIM lacks pathognomonic features on conventional imaging and is often mistaken for more common dural-based lesions such as meningioma or schwannoma. In our case, the well-circumscribed, homogeneously enhancing extra-axial lesion initially favored a benign dural neoplasm. However, advanced MRI sequences provided subtle clues. The marked hyperintensity on T2-weighted and FLAIR images likely reflects the high-water content of the gelatinous myxoid matrix, a key histological hallmark of myxomas [5,11]. Although nonspecific, this finding, together with minimal perilesional edema and the absence of diffusion restriction (indicative of low cellularity), supported a benign, slow-growing mesenchymal process.

The differential diagnosis of PIM includes several other myxoid and dura-based lesions, each with distinct diagnostic features. Myxoid meningioma, a rare histological subtype, shares the myxoid stroma but typically exhibits characteristic meningothelial whorls, psammoma bodies, and epithelial membrane antigen (EMA) positivity on immunohistochemistry—features conspicuously absent in PIM [5]. Chordoid meningioma presents with a chordoid appearance and often shows lymphoplasmacytic infiltration, which is not seen in pure myxomas. Low-grade fibromyxoid sarcoma is a more aggressive lesion characterized by a swirling growth pattern, alternating fibrous and myxoid areas, and the presence of MUC4 expression, which helps differentiate it from PIM [12]. Myxoid liposarcoma and extraskeletal myxoid chondrosarcoma are also considerations but exhibit distinct cytogenetic and immunohistochemical profiles. In practice, the combination of imaging features (T2 hyperintensity, homogeneous enhancement, no diffusion restriction), histopathological findings (stellate cells in a myxoid matrix with no atypia or mitoses), and exclusion of a cardiac source is sufficient to establish the diagnosis of PIM.

Histopathological examination remains the diagnostic cornerstone. In our case, microscopic evaluation revealed the characteristic features of a myxoma: stellate and spindle-shaped cells embedded within an abundant, sparsely cellular, myxoid matrix, surrounded by a fibrous capsule [7,11]. Special stains—Masson’s trichrome highlighting collagen and Gomori’s reticulin stain demonstrating a delicate supporting network—provided confirmatory evidence and helped differentiate PIM from other myxoid neoplasms such as myxoid meningioma or low-grade sarcoma.

A critical step in diagnosing PIM is the rigorous exclusion of a cardiac primary source, given the profound differences in prognosis and management. Cerebral lesions secondary to cardiac myxoma carry risks of recurrence, additional embolic events, and require systemic cardiac management [4,7]. In contrast, PIM typically follows a benign course after complete resection. In our patient, comprehensive cardiac evaluation with transthoracic echocardiography and cardiac MRI revealed no intracardiac mass, confirming the diagnosis of PIM and obviating the need for long-term cardiac surveillance.

Therapeutic management centers on gross total surgical excision, which is curative in most cases [2,5,[12], [13], [14], [15]]. In our patient, meticulous microsurgical dissection via a pterional approach allowed complete removal of the well-capsulated tumor. The postoperative course was uneventful, and at 18-month follow-up, the patient remains seizure-free with no radiological evidence of recurrence. This excellent outcome underscores that complete resection is associated with a favorable prognosis and a low risk of recurrence [5,14].

## Conclusion

**Fig. 4:**
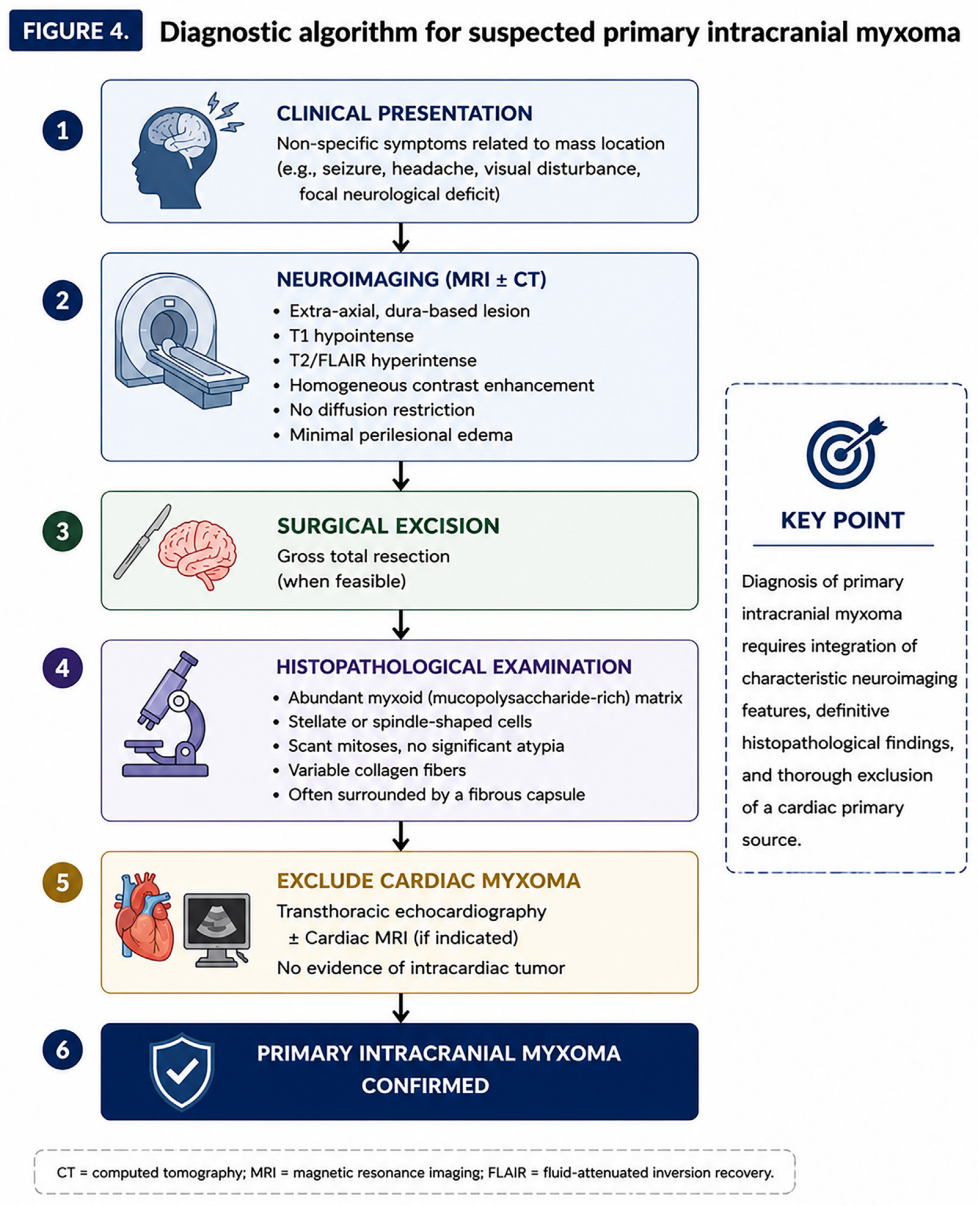
**Proposed diagnostic algorithm for suspected primary intracranial myxoma.** The algorithm outlines a systematic approach to the diagnosis of primary intracranial myxoma, integrating clinical presentation, characteristic neuroimaging findings, histopathological confirmation following surgical excision, and exclusion of a cardiac primary source by echocardiography with or without cardiac magnetic resonance imaging. This multimodal pathway facilitates differentiation of primary intracranial myxoma from other dura-based and myxoid intracranial lesions.

This case highlights primary intracranial myxoma as a rare but important diagnostic consideration for well-circumscribed, dura-based lesions in children. Diagnosis requires a multimodal approach integrating suggestive neuroimaging findings, definitive histopathology, and thorough systemic investigation to exclude a cardiac source (Fig. 4). Complete surgical resection remains the treatment of choice and is associated with an excellent prognosis and low recurrence risk, as demonstrated in our patient's favorable 18-month outcome.

## Submission statement

This manuscript is original and has not been submitted elsewhere in part or in whole.

## Authors contribution

Moustafa A. Mansour contributed to study conception, surgical management, manuscript drafting, and critical revision. Reem W. Malaeb contributed to literature review, data collection, manuscript drafting, and formatting. Afnan Morad contributed to clinical data acquisition, imaging analysis, and manuscript editing. Rawad Hakim contributed to pathological analysis and manuscript review. Hamdi Nabawi Mostafa contributed to surgical assistance and clinical follow-up. Mohamed Said Elsanafiry contributed to patient management, intraoperative assistance, and manuscript review. All authors read and approved the final manuscript.

## Ethics approval and consent to participate

Ethical approval for this study (Ref# NIH_25_02136) was provided by the Ethical Committee NIH of Nasser Institute Hospital, Cairo, Egypt on 15 January 2025. The patient’s guardian provided a written informed consent to publish the case and any related data.

## Patient consent

The authors declare that they have obtained consent from the patient.

## References

[1] Kawatra M., Bhandari V., Phatak S., Kulkarni D. (2013). Primary occipital myxoma: a rare case report. J Pediatr Neurosci.

[2] Menon R.K., Goel A., Shah A., Goel N., Rajashekharan P. (2008). Primary intracranial myxoma of the parietal region. Illustrated case report. J Neurooncol.

[3] Ma K., Zhao D., Li X., Duan H., Yan C., Wang S. (2023). Case report: multiple brain metastases of atrial myxoma: clinical experience and literature review. Front Neurol.

[4] Lee V.H., Connolly H.M., Brown R.D. (2007). Central nervous system manifestations of cardiac myxoma. Arch Neurol.

[5] Graham J.F., Loo S.Y., Matoba A. (1999). Primary brain myxoma, an unusual tumor of meningeal origin: case report. Neurosurgery.

[6] Moiyadi A.V., Moiyadi A.A., Sampath S., Kalpana S.R., Mahadevan A., Shankar S.K. (2007). Intracranial metastasis from a glandular variant of atrial myxoma. Acta Neurochir (Wien).

[7] Charabi S., Engel P., Bonding P. (1989). Myxoid tumours in the temporal bone. J Laryngol Otol.

[8] Ipek G., Erentug V., Bozbuga N., Polat A., Guler M., Kirali K. (2005). Surgical management of cardiac myxoma. J Card Surg.

[9] Nagatani M., Mori S., Takimoto N., Arita N., Ushio Y., Hayakawa T. (1987). Primary myxoma in the pituitary fossa: case report. Neurosurgery.

[10] Klein M.V., Schwaighofer B.W., Sobel D.F., Hesselink J.R. (1990). Primary myxoma of the posterior fossa. Neuroradiology.

[11] Osterdock R.J., Greene S., Mascott C.R., Amedee R., Crawford B.E. (2001). Primary myxoma of the temporal bone in a 17-year-old boy: case report. Neurosurgery.

[12] Powers C.J., Pizzi C.C., Cummings T.J., Friedman A.H. (2006). Primary myxoma of the parafalcine meninges. Case report. J Neurosurg.

[13] Yin H., Cai B.W., An H.M., You C. (2007). Huge primary myxoma of skull base: a report of an uncommon case. Acta Neurochir (Wien).

[14] Oruckaptan H.H., Sarac S., Gedikoglu G. (2010). Primary intracranial myxoma of the lateral skull base: a rare entity in clinical practice. Turk Neurosurg.

[15] Ryu J.I., Cheong J.H., Kim J.M., Kim C.H. (2015). A primary ossifying intracranial myxoma arising from the ethmoid sinus. J Korean Neurosurg Soc.

